# Knowledge and attitudes of us adults regarding COVID-19

**DOI:** 10.1186/s12245-020-00309-6

**Published:** 2020-11-02

**Authors:** Christopher Hogan, Massud Atta, Paul Anderson, Tej Stead, Matthew Solomon, Paul Banerjee, Bryan Sleigh, John Shivdat, Amanda Webb McAdams, Latha Ganti

**Affiliations:** 1 Coliseum Medical Centers Emergency Medicine Residency Program, Mercer School of Medicine, Macon, GA USA; 2 Brown University, Providence, RI USA; 3 Envision Physician Services, Plantation, FL USA

**Keywords:** COVID-19, Attitudes, Social distancing, Vaccination

## Abstract

This was a survey of the general non-healthcare-worker USA population regarding their knowledge and attitudes toward the COVID-19 pandemic. Almost everyone practiced social distancing. Women were significantly more likely to be worried about contracting the virus than men (65% vs. 43%, *p* = 0.0272). There was also a linear trend with age, with older Americans being more worried about contracting the virus. Women were also significantly likely to have received the influenza vaccine this past season compared to men (60% vs. 37%, *p* = .0167). Similarly, women were significantly more likely to get the influenza vaccine next season than men (77% vs. 46%, *p* = .0014.). Overall, across every age group, geographic part of the USA and gender, more (or the same) Americans plan on getting the influenza vaccine next season compared to last, but not fewer. This may reflect more awareness of preventative health brought on by the COVID-19 pandemic.

## Introduction

In early 2020, few people outside of the medical community in the USA were concerned about SARS-CoV-2. However, the novel coronavirus had already triggered public health concerns in China and Europe and was beginning to spread throughout the rest of the world, including the USA [1, 2]. Other coronaviruses, namely SARS-CoV and MERS-CoV, had previously engendered public health concerns in the SARS and MERS outbreaks in 2003 and 2012, respectively. Following those epidemics, a bat reservoir in which a coronavirus had originated was identified as a potential source for future coronavirus outbreaks in March 2019 [3]. In March 2020, public perception rapidly changed when COVID-19, the disease resulting from SARS-CoV-2, became classified as a pandemic. Daily interactions with our patients transformed as the public began to adopt infection-limiting measures recommended by the CDC [4].

This survey attempts to gauge public perception and social habit modifications adopted during this pandemic. Reduction of *R*_0_ to approach zero, at least in the most vulnerable populations, became a widely accepted goal [5, 6]. Willingness by the public to take precautions like wearing a mask and social distancing has become essential in preventing the spread of COVID-19. Based on the general reception of CDC precautions since their implementation, it is supposed that the general public is willing to comply. Specifically, how these precautions are perceived by the public and willingness to follow these precautions will be explored in this survey.

All relevant data at the time of this survey indicated that the SARS-CoV-2 transmission rate was comparable to the seasonal flu, with numbers based on statistical models from monitored outbreaks [7, 8]. General precautions that could decrease the spread of seasonal flu are being used to decrease the spread of COVID-19. Thus, a secondary public health benefit from the COVID-19 precautions includes a likely decrease in seasonal flu transmission. Individuals who contract seasonal influenza are more likely to obtain a vaccine the following year [9]. Decreasing influenza transmission in the general population provides a reduction in morbidity and mortality to the at-risk populations from respiratory infections [10, 11]. Prevention of COVID-19, seasonal influenza, and other respiratory illnesses also reduces healthcare costs while increasing quality of life [12]. These are the focus of this public research survey.

## Methods

A cross-sectional survey of adults aged 18 and over in the USA was conducted in April 2020 using the Google Consumer Survey methodology to assess knowledge and perceptions of the COVID-19 pandemic. Google Consumer Surveys show questions across a network of premium online news, reference, and entertainment sites, where they get embedded directly into content, as well as through a mobile app, Google Opinion Rewards. On the web, users answer questions in exchange for access to that content, an alternative to subscribing or upgrading. The user's gender, age, and geographic location are inferred based on anonymous browsing history and IP address. On the mobile platform, users answer questions in exchange for credits for books, music, and apps, and users answer demographic questions when first downloading the app. Using this data, Google Consumer Surveys builds a representative sample. This sample is weighted to match univariate distributions of age, geographical region (Midwest, Northeast, South, and West), and gender. However, this weighted sample does not necessarily reflect the joint distributions (for instance, the proportion of women over 65 in the South) in the USA. Pearson’s chi-squared test of independence was used to analyze discrepancies in responses among certain demographic groups.

This Google Consumer Survey was administered in such a way as to garner a validated, representative sample regarding gender, location, and basic demographics of US adults. However, the responses were anonymous, and no protected health information was collected in an identifiable manner. The demographics were collected by the Google survey team, and the specifics were not known to the authors.

## Results

There were 113 participants in the survey. From the original 113 subject, 101 were found to be non-healthcare workers and were used for the further remaining survey questions. The study is focused on the responses from the 101 remaining subjects to explore what is known amongst non-healthcare workers. Of the 101 participants, 54 were men and 47 were women. In addition, there are demographic categories in ages and location. Before weighting, ages were divided into 18–24 years (24%), 25 34 years (28%), 35–44 years (18%), 45–54 years (9%), 55–64 years (6%) and 65+ years (16%). The geographic region was somewhat evenly split across the Midwest (25%), Northeast (24%), South (29%), and West (23%).

The following are the answers to the each of the survey questions, weighted by age, gender, and region.

### “How long do you think the social/economic effects of COVID-19 will last?”

This question reflects what participants project the length of time it would take to overcome the social and economic effects of COVID-19. 61.4% believed that it would last at least 6 months, with 38.6% believing it would last over a year. This is particularly appreciated in the Northeast, where 72.2% felt it would last at least 6 months, and 58.6% believed it would last over a year. The more devastating numbers of COVID-19 positive cases and mortality in the USA. at the time of the survey were in the Northeast (New York, New Jersey), which could explain the increased belief that the virus will last a long time.

### “Have you been practicing social distancing?”

The vast majority of respondents have been practicing social distancing, 91.1% of the respondents answering ‘Yes, almost always’ and 8.9% answering ‘Yes, sometimes.’ There was no respondent that answered ‘No’ to social distancing. However, what varies is the amount of ‘Almost always’ versus ‘Sometimes’ between regions. In the Northeast, 100% answered ‘Almost always’ while 97.3% of respondents from the West answered the same. In slight contrast, both Southern and Midwestern regions responded ‘Almost always’ 85.3% and 85.2%, respectively. This could be related to the severity of the outbreak in the West and Northeast or perhaps each region’s shelter-in-place practices compared to that of the South and Midwest.

### “Are you concerned about contracting the virus?”

**Fig. 1:**
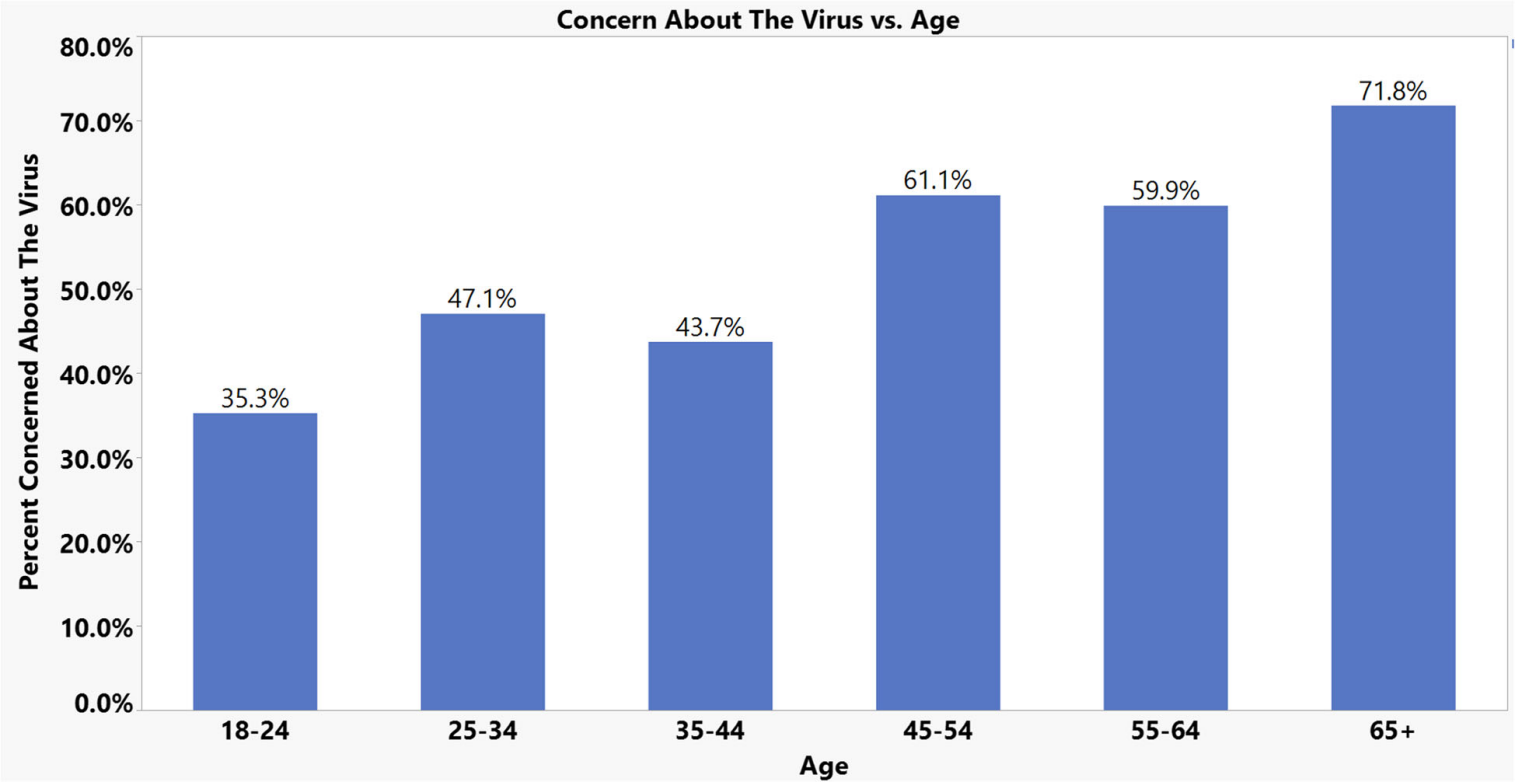
COVID-19 survey: Concern about the virs vs. age of respondent

Of the 101 respondents, the response to this question was relatively equal with 53.9% replying ‘Yes’ and 46.1% replying ‘No.’ There was relatively little variation between the Western (49.9%), Midwestern (51.9%) and Southern (51.1%) regions in the amount of people who were concerned about contracting the virus. However, 68% of Northeastern respondents were concerned about contracting it. In addition, 65.0% of female respondents were concerned about contracting the virus as opposed to 43.2% of male respondents, which was significant with a p-value of 0.0272. Respondents aged 65 and over were most concerned with the virus at 71.8% as compared to the remaining age groups. There appears to be a general trend of older Americans being more concerned about the virus (Fig. 1).

### “If you believe you have COVID-19 or have been exposed to it, you should:”

This question had an available multiple-choice component to it. Respondents could choose between: ‘Self-quarantine (stay at home)’, ‘Go to my doctor’, ‘Call my doctor’, ‘Go to Emergency Room.’ A majority of respondents (61.9%) answered that should ‘Self-quarantine (stay at home)’ with a 34.4% answering ‘Call my doctor.’ This trend was relatively the same for other demographics except for the 18–24-year-old age group where 55.8% of this age group answered, ‘Call my doctor’ and 31.7% answered to ’Self-quarantine (stay at home).’

### “What is the best way to prevent the spread of COVID-19?”

The answer choices provided for this question were: ‘Social Distancing,’ ‘Hydroxychloroquine,’ ‘Antibiotics,’ and ‘Flu Medicine.’ Of the 101 respondents, 99 answered that ‘Social Distancing’ was the best way to prevent the spread of COVID-19, with just two responding that hydroxychloroquine was most effective.

### “How severe is COVID-19 compared to the flu?”

The majority of respondents to this question answered that COVID-19 is more severe (73.1%) or as severe (22.3%) compared to influenza, whereas 4.6% indicated it was less severe. This trend was relatively equal across gender, age groups, and regions. It appears, from this sample, that COVID-19 is considered more severe than influenza in the majority of the population.

### “If a vaccine becomes available for COVID-19, would you get it?”

Participants of the study were allowed to answer between ‘Yes’ and ‘No’ when responding to this question. The majority of respondents (74.1%) answered that they would get the COVID-19 vaccine if it is available. This was similar across regions and gender. However, amongst the age groups, participants aged between 45–54 years preferred not to receive the vaccine (42.6%), which is the opposite compared to all other age groups.

### “Did you get the flu vaccine this year?”

Of the 101 participants, 52% received their flu vaccine this year. When comparing male and female participants, the response was drastically different. 60.3% of women received their flu vaccine as opposed to 36.5% of men. This difference was significant with *p* = .0167. The Northeast region reported the highest amount of flu vaccinations at 65.3%. In addition, the age group with the highest percentage of flu vaccines this year was 55–64 years (66.4%) while the 25–34-year-old group was the least (31.4%).

### “Do you plan to get a Flu (influenza) vaccine next year?”

The majority of respondents (61.4%) planned to receive the influenza vaccine next year. Regionally, the responses have been in favor of planning to receive the influenza vaccine next year with the Northeast being the highest (75.5%) rate of positive responses and the South being the lowest (55.1%). However, amongst the age groups there were groups that still do not feel the need for influenza vaccinations. The age groups 25–34 years (43.7% planning to receive vaccine next year) and 45–54 years (42.5%) had a majority of respondents who did not plan to receive the vaccine next year. 77.3% of women planned to receive the vaccine next year, compared with 46.3% of men. This difference was significant with *p* = .0014.

### Side-by-side comparisons of participants who received their Influenza vaccination compared to next year

**Fig. 2:**
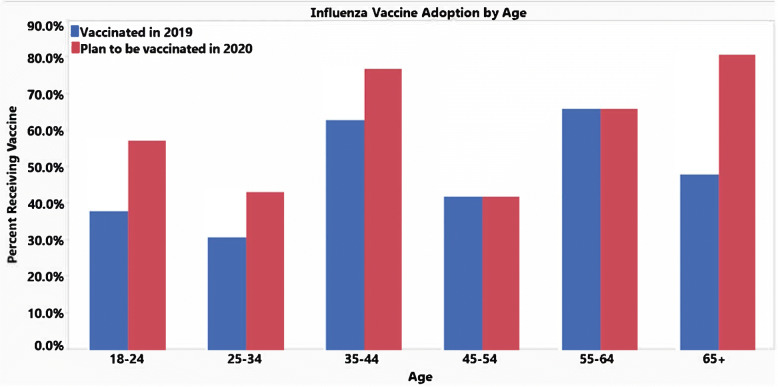
Influenza vaccine adoption by age

**Fig. 3:**
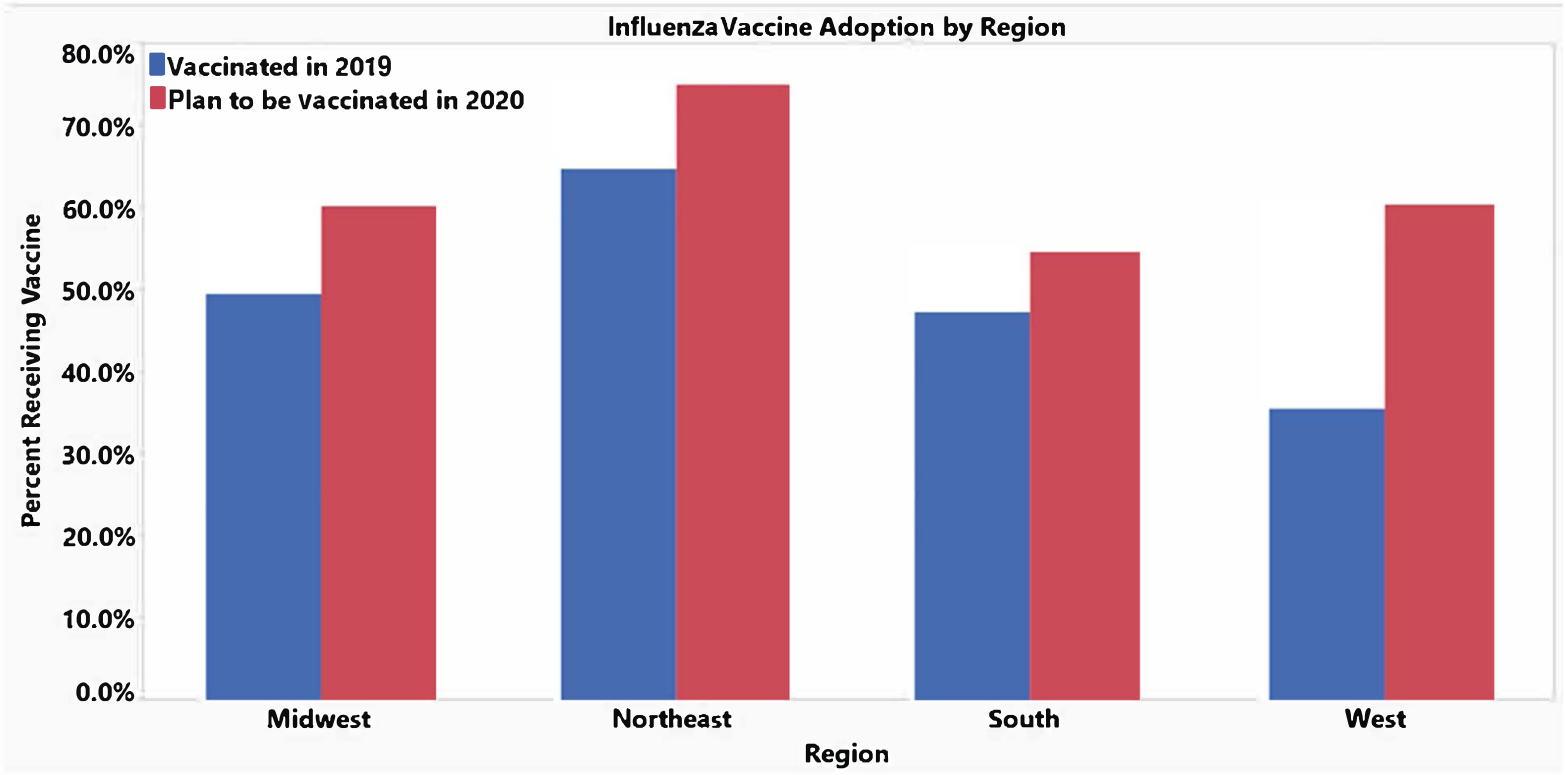
Influenza vaccine adoption by region

**Fig. 4:**
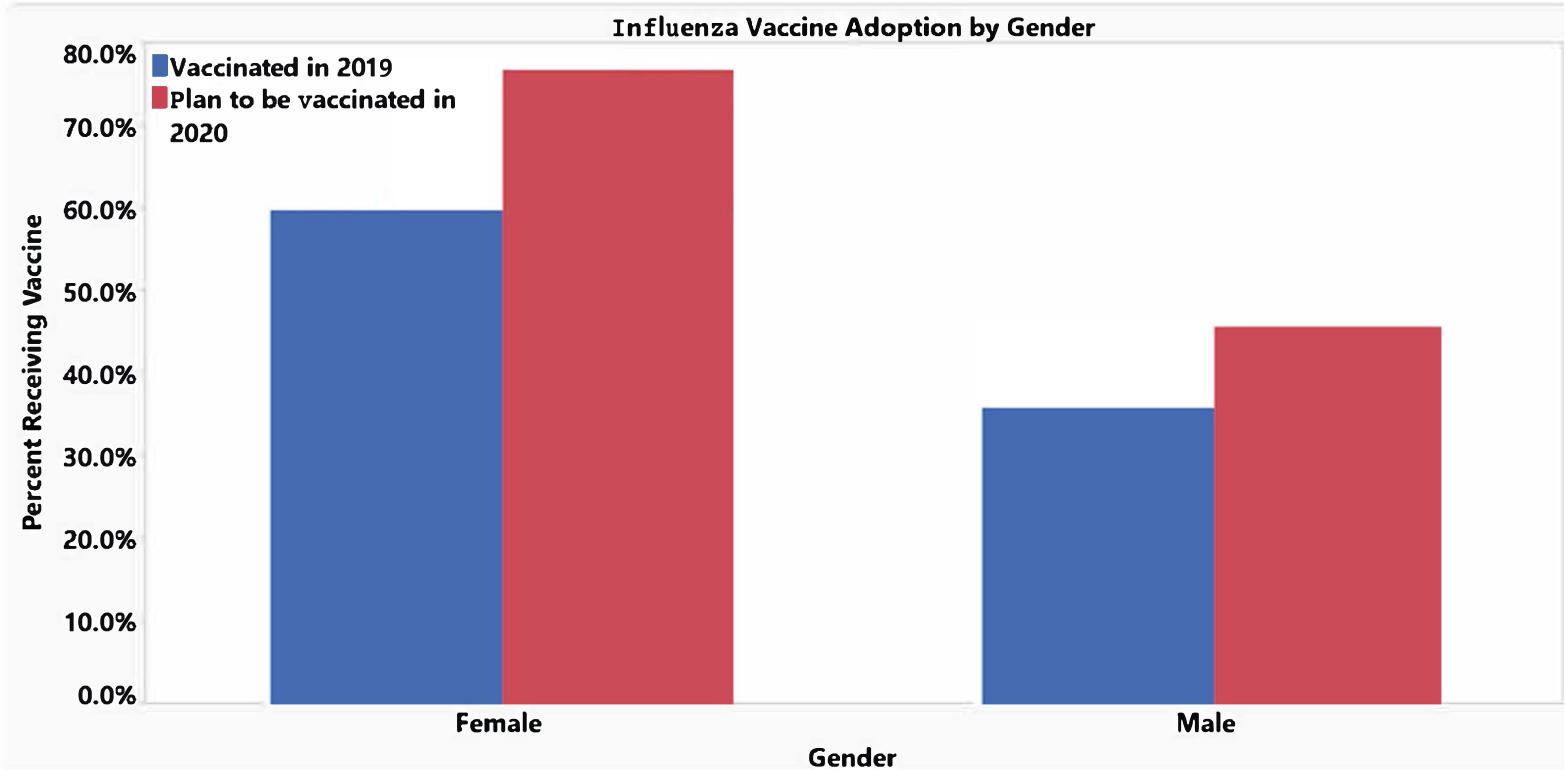
Influenza vaccine adoption by gender

These next set of graphics compare the responses for the influenza vaccine from this year and the respondents’ plans to receive the flu vaccine next year. Generally, the trend is that respondents are more inclined to receive the flu vaccine next year from 48.2% ‘Yes’ responses this year to 61.4% ‘Yes’ responses next year. It is possible the COVID-19 pandemic has made it more encouraging for people to consider being vaccinated as a preventative measure for battling the seasonal influenza. With the exception of the age groups 45–54 and 55–64, there is an increased number of respondents willing to receive the influenza vaccine next year (Fig. 2). This upwards trend is seen regionally especially in the West and Northeast, which had significant jumps in favor of receiving the influenza vaccine (Fig. 3). This could be related to the COVID-19 pandemic having more devastating effects in these regions compared to the Midwest and Southern regions. In addition, between genders, there is an increase in both male and female participants who are in favor of receiving the influenza vaccine next year (Fig. 4). However, it still appears that women are consistently more likely to receive the influenza vaccine. The proportion of people receiving an influenza vaccine next year has either stayed constant or increased in every single demographic, which indicates that the COVID-19 pandemic has overall very strongly increased people’s awareness of the benefits of vaccination.

## Discussion

The COVID-19 pandemic has affected the world population and changed the way we think about communicable diseases. Though, the extent of this pandemic will not be fully realized for some time. Currently, the USA is being affected in its social, economic, and health arenas. This are best illustrated by social distancing practices, restricted travel, furloughs and business closures, as well as self-quarantine and increasing PPE requirements for the general population. There has not been a novel illness to affect the world like this since H1N1 in 2009, and government intervention did not come close to the same level as it has during the COVID-19 pandemic.

We specifically chose to survey non-healthcare workers to assess knowledge and attitudes of the general population. Most believe the effects of COVID-19 will last at least 6 months. Although most respondents also reported almost always practicing social distancing, most are still concerned they will contract the virus. Respondents in the Northeast and West are most inclined to think that the COVID-19 pandemic will last at least 6 months and believe in social distancing practices. Also, respondents in the Northeast particularly believe that COVID-19 is a concern. This is likely an outcome of these regions having more COVID-19 cases than the rest of the country at the time of this survey. Generally, COVID-19 is perceived to be a high-level threat and appears to be realizing that perception as further testing and reporting continues to report increasing deaths. Most respondents agreed that self-quarantine and social distancing were the best ways to avoid the spread of the virus, which have successfully flattened the curve in most areas although some parts of the country have seen recent increases in cases. This parallels with expert opinions at the time of this survey.

The respondents’ perception of the current COVID-19 pandemic as a high-level threat will likely translate to a greater openness to getting vaccinated. Most respondents state they believe COVID-19 is more severe than the flu and if a vaccine were available, they would receive it. The respondents were also asked if they received the flu vaccine in the last year and about half stated they did not. It appears that increased awareness of communicable diseases and high threat perception has led most respondents to state they will receive a flu vaccine in the future. Specifically, we found results that may suggest a trend of women both being more concerned about contracting the virus and being more likely to receive an influenza vaccine this year, as well as next year. This may reflect a trend of women being more likely to be aware of public health safety measures. We noted, however, that men and women are nearly equally likely to engage in social distancing. This may be due to the widespread adoption of the practice in combination with government shelter-in-place orders.

## Conclusion

Due to the high perceived threat of COVID-19, most respondents are now more aware of communicable diseases, prevention practices, and the potential effects of illness on the community. It is possible that the change in health perception will lead to an increase in seasonal influenza vaccination.

## Data Availability

The datasets used and/or analysed during the current study are available from the corresponding author on reasonable request.

## Abbreviations

ED: Emergency department
SARS: Severe acute respiratory syndrome
MERS: Middle Eastern respiratory syndrome
CDC: Centers for Disease Control
